# COVID-19 Positive Stroke Patient With Large Vessel Occlusion in the Epidemic

**DOI:** 10.7759/cureus.19848

**Published:** 2021-11-23

**Authors:** Mustafa Cetiner, Güngör Çakmakçı, Muhammed Alperen Bardakçı, Gönül Akdağ, Sibel Canbaz Kabay

**Affiliations:** 1 Department of Neurology, Kütahya Health Sciences University School of Medicine, Kütahya, TUR

**Keywords:** thrombectomy, stroke, infarct, large vessel occlusion, covid-19

## Abstract

COVID-19 disease causes various neurological disorders. Of these, stroke is the most devastating and difficult to manage in epidemic conditions. An increase in the rate of acute ischemic stroke in hospitalized coronavirus patients and stroke with large vessel occlusion due to COVID-19 disease have been reported in recent publications. The management of these patients is difficult and becomes even more challenging in epidemic conditions. A 71-year-old man suddenly developed left-sided weakness while he was hospitalized for COVID-19 disease. Cerebral computed tomographic angiography showed a terminus of the right internal carotid artery. The occluded vessel was completely recanalized by endovascular therapy. Left-sided hemiparesis resolved completely. As a result of this study, cryptogenic stroke was considered in the etiology of stroke. In this report, we present a case of stroke with COVID-19, who developed large vessel occlusion accompanied by splenic infarction while hospitalized due to COVID-19 disease and was successfully treated with endovascular thrombectomy under epidemic conditions.

## Introduction

COVID-19 disease causes various neurological disorders. Among these, stroke is the most disabling illness and its management is difficult during a pandemic [1-3]. Although the pathogenic mechanism underlying the cerebrovascular complications caused by COVID-19 is not clear, it is hypothesized that severe acute respiratory syndrome coronavirus-2 (SARS-CoV-2) triggers the hyperinflammation process by binding to the angiotensin-converting enzyme-2 receptor on the surface of smooth muscle cells in the arterial endothelium. Also, the cytokine storm caused by hyperinflammation leads to cerebrovascular complications with coagulopathy and endothelial dysfunction. It has been shown that SARS-CoV-2 causes a 7.6-fold increase of cerebrovascular complication risk compared to the influenza virus [1,4]. Acute ischemic stroke (AIS) occurs at a rate of 1.3-5% in hospitalized coronavirus disease patients [2,3,5-7]. This rate is even higher in intensive care patients [1]. The results reported in the literature indicate that ischemic strokes with COVID-19 are associated with worse functional outcomes and higher mortality than non-COVID-19 ischemic strokes. The younger age of COVID-19 patients with stroke, relatively high severity of a stroke, and higher incidence of large vessel occlusions (LVO) suggest a causal relationship in terms of thrombosis susceptibility [4]. More cardiovascular events and multiple organ involvement are seen in these patients during hospitalization. Therefore, they have higher mortality rates [2].

In this case, we report a case of COVID-19, who presented with multiple thromboembolic complications involving the brain and the spleen, which was successfully treated by endovascular thrombectomy method under epidemic conditions.

## Case presentation

A 71-year-old male patient with hypertension using regular antihypertensives was receiving inpatient treatment at our hospital's pandemic clinic for COVID-19 pneumonia. His reverse-transcriptase polymerase chain reaction (RT-PCR) was positive in the nasopharyngeal swabs. The patient, who was conscious during his stay in the pandemic ward, was mobilized without support. Subfebrile fever was detected on the first day of the patient's follow-up. Respiratory rate was 24 breaths per minute, heart rate was 100 beats per minute, and oxygen saturation level was in the range of 88-90%. He needed intermittent nasal oxygen support. No additional pathology was detected in the physical examination. During his follow-up, favipiravir 1200 mg/day [per os (po)], amlodipine 10 mg/day (po), enoxaparin sodium 0.6 mL/day [subcutaneous (sc)], ipratropium bromide and salbutamol 0.5 mg (four times a day inhaler), paracetamol 1500 mg/day (po), acetylcysteine ​​600 mg/day, and moxifloxacin 500 mg/day (po) treatments were applied. Laboratory findings were C-reactive protein (CRP): 63 mg/L, white blood cell: 9.3, alanin aminotransferase: 113 U/L, aspartate aminotransferase: 95 U/L, D-dimer: 930 µg/L. In the pandemic clinic, on the 15th day, the patient was noted to develop right gaze deviation, left facial and hemibody weakness, and speech changes. The National Institutes of Health Stroke Scale (NIHSS) score was 13. The Alberta stroke program early CT score on non-contrast cranial CT was 10. CT angiography showed terminus of the right internal cerebral artery (ICA) and excellent collateral flow. The right anterior cerebral artery was filling from the opposite side. Souza collateral grading score was two in the right middle cerebral artery (MCA) [8]. Hyperintensity at the level of the right basal ganglia, suggesting a hyperacute infarction, was detected on diffusion-weighted imaging (DWI). The patient with marked clinical-radiological imaging mismatch was taken to the interventional neuroradiology angiosuite. Appropriate personal protective equipment (PPE) was worn before the procedure. Under sedo-analgesia, two pass thrombectomy was performed under aspiration through the Sofia 6F catheter (Microvention, California, USA). The right carotid artery was fully recanalized within 4.5 hours of the patient's clinical presentation [modified treatment in cerebral ischemia (mTICI) grade 3].

**Figure 1:**
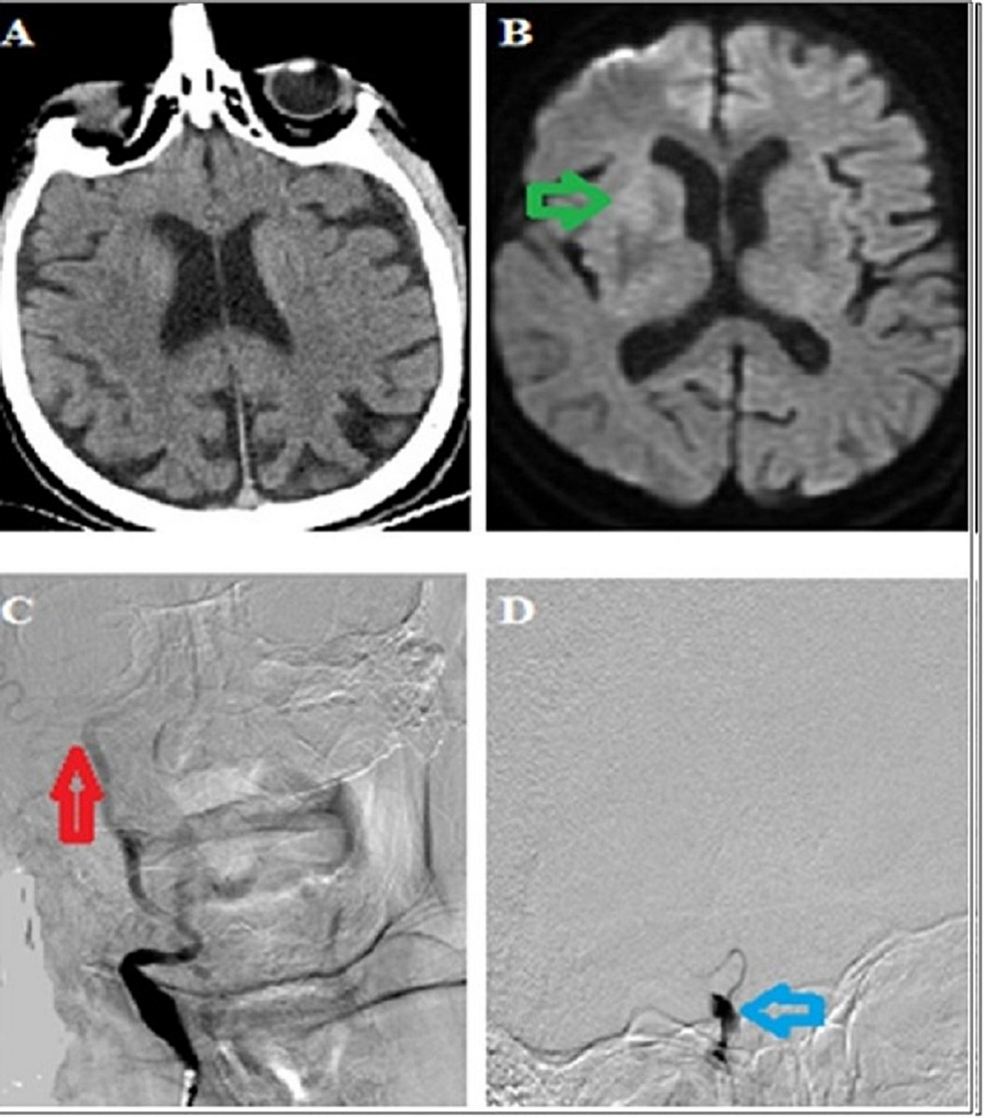
(A) The Alberta stroke program early CT score was 10 on non-contrast cranial CT 2.5 hours after symptom onset. (B) Hyperintensity at the level of the right basal ganglia in diffusion-weighted imaging suggesting a hyperacute infarction (green arrow). (C) and (D) Slow flow and carotid T occlusion in right internal carotid injection (red and blue arrow).

**Figure 2:**
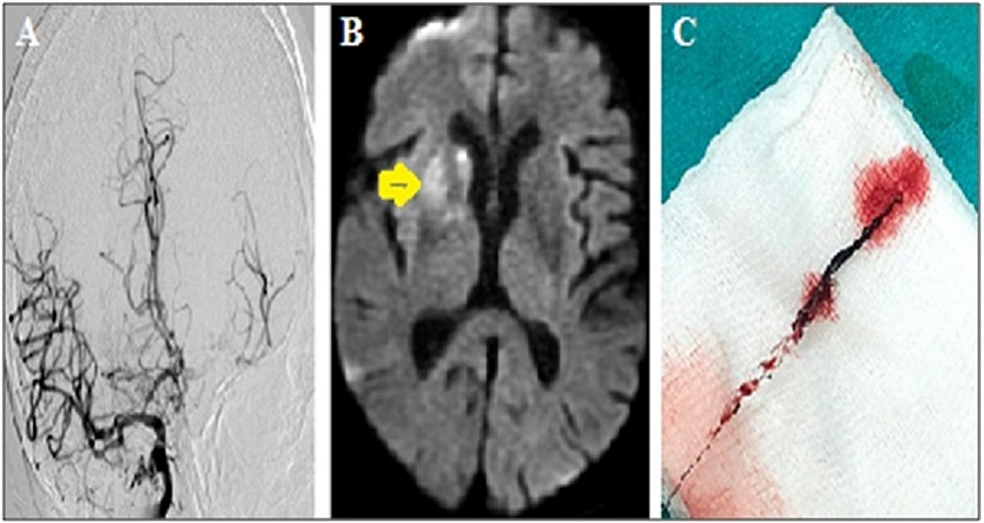
(A) Complete recanalization of the right carotid T occlusion (mTICI grade 3). (B) Acute infarction in the right basal ganglia (yellow arrow) in control diffusion-weighted imaging at 24 hours after the procedure. (C) The stent retriever and the thrombi. mTICI: modified treatment in cerebral ischemia.

Neuroimaging characteristics of the patient before and after treatment were presented in Figure 1 and Figure 2, respectively. After the procedure, the patient was followed up in the neuro-COVID intensive care unit. MRI brain performed after 24 hours of the clinical presentation showed an acute infarction at the level of the right basal ganglia. Significant improvement was observed in the patient's neurological examination. NIHSS score decreased to five. The patient could be mobilized without assistance. Transthoracic echocardiography and 24-hour rhythm Holter examinations were normal. Cryptogenic stroke was considered as the etiology. Two days after mechanical thrombectomy, the patient's state of consciousness completely recovered. During his clinical follow-up, he complained of abdominal pain localized to the left upper quadrant, nausea, and vomiting. Abdominal examination revealed tenderness in the left upper quadrant with palpation. Splenic infarction was also noted on the total body CT of the patient. In follow-up, the patient remained stable, and the control RT-PCR test was negative. The patient was discharged home on the fifth day of neuro-COVID intensive care hospitalization. Two weeks later, the patient applied to the neurology outpatient clinic for a control examination. In his neurological examination, his consciousness was normal, his muscle strength was full, and he could be mobilized without support. No pathology was detected in the physical examination.

## Discussion

Cases of LVO complicate the management of acute stroke management. In addition, the treatment of acute stroke treatment is more difficult due to the pandemic conditions. This stroke case, which presented with LVO triggered by the COVID-19 disease, was successfully treated with mechanical thrombectomy, taking into account the principle of "time is brain," despite the difficulties in the pandemic conditions. This case provides information on the characteristics and treatment management of stroke patients with COVID-19. Current guidelines recommend mechanical thrombectomy for the treatment of patients with ischemic stroke caused by occlusion of the internal carotid artery or proximal middle cerebral artery and who have significant neurologic deficits [9].

This 71-year-old male patient with a severe stroke clinic had only hypertension as a stroke risk factor. In addition, liver function tests and D-dimer levels were high. LVO is common in COVID-19 patients presenting with AIS [1,3]. The severity of stroke is high and most are young male patients with cryptogenic stroke. Vascular risk factors are low. Concomitant systemic thrombosis is not uncommon. They cause poor functional results and high mortality [1,3,4]. Even in patients who underwent thrombectomy and achieved successful recanalization, poor functional outcomes have been reported in the short-term follow-up [3]. According to the study of Li et al., cerebrovascular events were more common in elderly COVID-19 patients with risk factors such as hypertension and diabetes mellitus [7].

SARS-CoV-2 is a potentially higher trigger for acute ischemic stroke, possibly through immune-mediated coagulopathy [1]. This case was evaluated as major vascular occlusion triggered by COVID-19 disease.

Multiple systemic thrombosis, such as venous thrombosis, splenic embolism, and pulmonary embolism, may accompany COVID-19 patients [1]. In this case, cerebral LVO was accompanied by splenic infarction.

In most COVID-19 patients presenting with stroke, signs of stroke occur within the first 21 days after the onset of COVID-19 [1]. Laboratory findings such as elevated D-dimer, lactate dehydrogenase, and liver enzymes concentration are seen in these patients [1,10]. During the epidemic, mechanical thrombectomy procedures, such as the door-groin puncture time, were prolonged. However, it is emphasized that the clinical results have not changed in the studies, therefore, mechanical thrombectomy should be performed even during the epidemic [1,11]. This patient developed a stroke 15 days after the onset of COVID-19 symptoms. Our door to groin puncture time was approximately 90 minutes. Successful recanalization was achieved 4.5 hours after the onset.

It has been reported that cerebral LVO may be in the form of multiple vessel occlusion [12,13]. In this case, there was only right ICA terminus occlusion and infarct was observed only in the basal ganglia. There was no other vascular involvement.

## Conclusions

We should approach patients with COVID-19 stroke with the principle of "time is brain." Endovascular thrombectomy is very effective in selected patients with AIS with LVO. It should be considered that COVID-19 may be an important risk factor for the development of stroke and LVO, and patients' access to thrombectomy should be preserved, even under epidemic conditions.
